# “Social learning, innovation, and sustainability: The search for directions beyond a systematic literature review”

**DOI:** 10.1016/j.heliyon.2024.e28431

**Published:** 2024-03-27

**Authors:** Marcos da Silva-Jean, Jordana Marques Kneippb

**Affiliations:** a Professor at the Federal Institute of Education, Science, and Technology (IFSul), Av. das Indústrias, 865, Universitário, Venâncio Aires, RS, 95800-000, Brazil; b Professor at the Graduate Program in Administration at the Santa Maria Federal University, Av. Roraima, 1000, Prédio 74 C, Cidade Universitária, Santa Maria, RS, 97105-900, Brazil

**Keywords:** Sustainability, Emancipatory learning, Double-loop learning, Sustainable innovation, Systematic literature review

## Abstract

The literature frames social learning as a critical concept when searching for sustainability and innovation. Its prominent position in various studies has raised questions about the role played by such a theory and the possibility of new research perspectives. Therefore, this paper analyzed future research directions in social learning, sustainability, and innovation through a systematic literature review. By using four guiding questions, this qualitative study conducted a systematic literature review in the Web of Science, Scopus, and Scientific Electronic Library Online (SciELO) databases using the search terms “social learning,” “sustain*,” and “innovation.” to identify, evaluate, and interpret relevant available studies. For the analysis, hermeneutic units were created to construct relationships between papers of different perspectives and subjects, namely social learning for sustainability and innovation. The results demonstrated that instrumentalizing social learning theory has been essential to promoting sustainability and innovation. Regarding innovation, social learning creates opportunities for innovation because it motivates change and questions rules and norms. Studies on social learning for sustainability were the most representative although few studies have focused on what individuals become after an intervention based on social learning; they only applied the concept and demonstrated the processes of carrying out interventions, who participates, and how and why it happens. Additionally, the highlighted changes are not ontological, but epistemological, even though social learning and innovation seem to intertwine to change and promote sustainability. This study's contribution lies in the theoretical perspective since a kind of taxonomy was produced with the results from the databases, enabling advances for future research. The uniform treatment attributed to innovation was a limitation, refraining from differentiating its various nuances. Nevertheless, the originality of this article is the intersection between two theoretical perspectives — social learning and innovation — in the common and complex field of sustainability.

## 1 Introduction

Studies on sustainability have aroused the interest of various areas of knowledge, leading to different explanations of environmental challenges through numerous theories [1]. Consequently, the theoretical concepts of social learning and innovation theory emerge, sometimes acting interconnectedly to explain sustainability phenomena [[2], [3], [4]]. Mouleart [5] advocated for moving different theories around a common problem, and Neumeier [6] highlighted the role of collaborative actions in discussing social aspects. Global organizations, such as the Organisation for Economic Co-Operation and Development (OECD) [7], have been indicating that the world needs a guideline; otherwise, exploiting natural resources, deteriorating social rights, and increasing economic inequalities that have compromised the living standard of populations can intensify [8].

Social learning is a change in understanding reflected in wider social units through social interactions from person to person in a social network [9]. This concept arises from cognitive psychology [10], especially after the “John the Wolf experiment” conducted by Bandura [11,12], who concluded that imitating and observing others influence social learning, which are critical aspects of the theory. However, Bandura [11] focused on individual learning, even though the “Bob-John experiment” took place in a social context [9].

Therefore, social learning overcomes individual understanding by involving social change, mutual learning, and benefits to broader social-ecological systems since learning demands social interaction [9]. The message needs to be spread from person to person through a social network, enabling the individuals involved to negotiate rules and norms as bearers of multiple experiences based on common sense or scientific knowledge [9]; the message circulates both via conversation and mass media.

Another aspect of social learning is addressed by Argyris and Schon in organizational contexts [13]. Learning happens in single and double-loops; in the first case, the individual is inspired by images constructed from their experience and acts within known norms, whose behavior pattern does not change the organization's status quo. The single-loop learning is a *sine qua non* condition for double-loop learning to occur in organizations, such as industrial companies, which are engaged with internal and external environments when seeking to correct errors aiming at effectiveness, including the best way to achieve goals and objectives within pre-existing norms [13].

Nevertheless, in certain contexts, solving errors is so complex that organizational norms are questioned, including inserting new technology to change how managers, workers, and members adopt new marketing, administration, and production approaches. Changes under an effectiveness imperative often create a conflict in the organizational norms that cannot be resolved through single-loop learning because it is rooted in established rules and habits that prevent the organization from achieving effectiveness [13].

The result demands restructuring organizational norms that must be incorporated into images and maps that encode individuals’ actions in organizations. This process is called a double-loop since there is dual feedback when detecting errors in the strategies that seek to improve effectiveness and, in the norms, defining effective performance. Evidence has shown that there may be collaboration through interaction between different actors (i.e., employees, managers, and government) in the single loop [13]. Nonetheless, a bigger change is required to reach patterns of norms and habits to set up a double loop, where there are conflicts between different members and groups within the organization. The consequence is learning and resolving these confrontations through new understandings [13].

While we notice adjustments in actions to achieve effectiveness in single-loop learning, an individual changes the organizational norms and practices in double-loop learning, causing structural questioning and requiring other changes from an action-reaction loop to reflect on the situation in a wider context. The definition of social learning corroborates Argyris and Schon's double-loop concept [13], as suggested by Reed et al. [9].

Hence, the simple interaction between different stakeholders as a condition for social learning is naive, especially regarding natural resource management. Simple interaction and holding workshops can be considered methods for pursuing social learning, although it is not exactly social learning [9]. The challenges of implementing sustainability require changes in behavioral patterns, such as those reported by Argyris and Schon [13].

The trajectory of research on innovation to mitigate environmental crises has reached sufficient maturity, leading to the emergence of innovation for sustainability — InovSus. It can introduce new or modified practices into production processes, technologies, products, or organizational systems to reduce environmental damage [14]. InovSus is essential for organizations to achieve the sustainable development goals set by the United Nations since it depends on innovation, being at least relevant [[14], [15], [16], [17], [18]].

Another terminology widely used as a synonym is “sustainability-oriented innovation” or “innovation for sustainability.” Klewitz and Hansen [19] affirmed that the term includes ecological and social aspects of products, processes, and organizational structures. The word “oriented” highlights that sustainability is not an end point but an uncertain direction since the environmental and social impacts of sustainable innovations are highly undetermined [20]. InovSus refers to a systematic attitude of a company in terms of economic, social, and environmental aspects, which implies that it cannot be understood through isolated actions; otherwise, the terminology “sustainable innovation” becomes inappropriate, as in the case of developing new environmentally responsible products [21].

Researchers have argued that sustainable innovation can benefit organizations in various ways, such as improving social image and profit performance [21,22]. Despite studies demonstrating a relationship between sustainable innovation and improved business performance, proving that sustainability-oriented practices can reduce environmental challenges, Gupta et al. [23] identified barriers to implementation, including the lack of policy frameworks and specific strategies from companies.

According to the literature [20], sustainability-oriented innovation is challenging for the organization because it demands changes in structures, philosophies and values, products, processes, and business practices to create social, environmental, and economic value. Nevertheless, not all organizational values promote sustainability-oriented innovation [24]. Therefore, it is necessary to use strategies that seek to evolve organizational values aiming at innovation-oriented sustainability.

Hence, InovSus is more disruptive than other innovations, requiring a multi-stakeholder approach as a collaborative practice [[25], [26], [27]]. It does not necessarily occur as a technological change but may relate to modification in processes, models, or business values [28].

This historical context makes searching for alternatives in different areas of knowledge for mitigating these problems highly relevant. Social learning, a process of change in individuals understanding to achieve social units within a community of practice, and innovation, an intervention oriented towards structural changes in a social dimension to systemically improve societies, are included [9,29]. Analyzing environmental projects indicates that social learning is an important aspect of managing crises in initiatives involving climate issues [30].

Although social learning and innovation focus on sustainability, studies are inadequate or incomplete. These concepts are related to each other, converging on environmental issues or social concerns, although the research field integrating both definitions to investigate sustainability issues has been explored little. Little is known about the relationship between social learning and innovation for sustainability, which is an opportunity for new studies to be developed, whose objective may be to understand how these concepts have been described in the literature.

The relevance of this article lies in investigating such relationships to prospect new research, and we sought to describe concepts and find patterns in the literature to support studies on the subject. Therefore, the question is: what are the future research directions in social learning, sustainability, and innovation from a systematic literature review?

The results demonstrated that instrumentalizing social learning theory has been essential to promoting sustainability and innovation. Few studies have focused on what individuals become after an intervention based on social learning; they only applied the concept and demonstrated the processes of carrying out interventions, who participates, and how and why it happens. Additionally, the highlighted changes are not ontological but epistemological, even though social learning and innovation seem to intertwine to change and promote sustainability.

This article is structured in five sections. First, the fundamental concepts are addressed, followed by the methodological procedures explaining the stages used for the literature review. The results and discussions are then presented, and several considerations are made to indicate the prospects for future research.

These concepts come from a study based on a systematic literature review, and the methods are described below.

## 2 Methodological procedures

This study employed a systematic literature review to analyze conceptual and empirical papers on social learning, innovation, and sustainability. Kitchenham's [31] definition of a systematic literature review is at the core of this article, as we sought to identify, evaluate, and interpret relevant available studies to answer the research question.

### 2.1 Search terms and selection criteria

We collected studies from the Scopus, Web of Science, and SciELO databases using the search terms “social learning,” “sustain*,” and “innovation.” The result files were exported in BIBtex or RIS format and imported into the State of the Art software through Systematic Review – Start (This is a software developed by the Software Engineering Research Laboratory of the Federal University of São Carlos - UFSCar to help the researcher on applying systematic literature reviews). Therefore, planning, selecting, and extracting information was only performed in the software, and the content analysis was conducted in the Atlas.ti software (this is a content analysis software widely used in qualitative research, developed in 1989 by Thomas Muhr [32] in Germany).

**Fig. 1:**
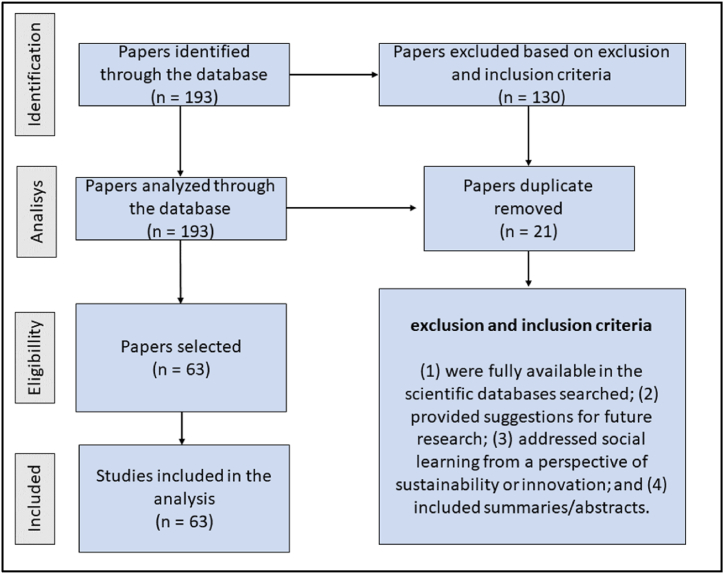
Flow chat of inclusion and exclusion criteria.

**Source:** Survey data.

The search returned 193 articles, and 63 remained after the inclusion and exclusion criteria — (1) were fully available in the scientific databases searched; (2) provided suggestions for future research; (3) addressed social learning from a perspective of sustainability or innovation; and (4) included summaries/abstracts — were established and judged by the authors separately and individually. We selected papers that simultaneously were fully available in the scientific databases, presented suggestions for future research, addressed social learning from a sustainability or innovation perspective, and had abstracts. The flow of the process of identification, selection, inclusion, and exclusion of articles can be visualized in Fig. 1.

We evaluated the papers’ quality, observing the criteria of textual coherence and cohesion, the objectivity of the methods and techniques reported, the appropriateness of the keywords, at least one citation in the databases, and the search terms in the title. The quality assessment may have been influenced by selection bias, where the experiences of the review authors may have impacted the choice of articles, as cautioned elsewhere [33,34]. How the analysis and interpretation occurred to systematize the knowledge of the selected articles are presented below.

### 2.2 Analysis and interpretation of the studies

**Fig. 2:**
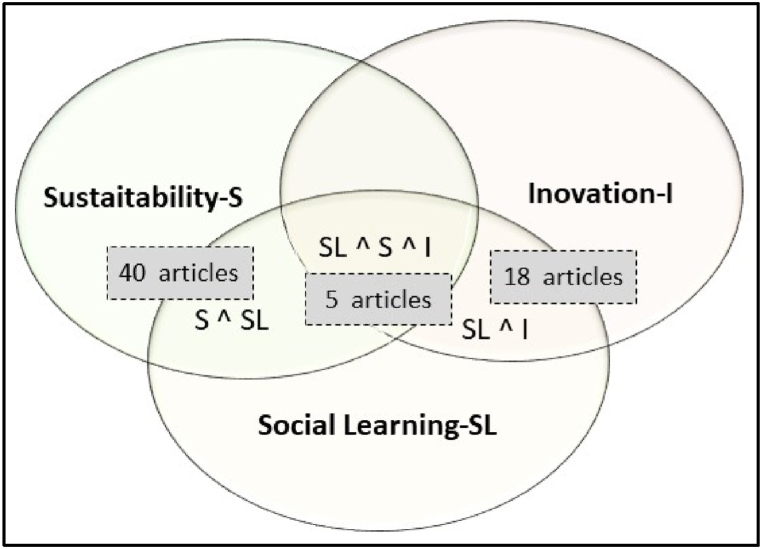
Distribution of the systematic literature review by research subject.

**Source:** Survey data.

The relationship between the studies contemplated by S ^ SL, SL ^ I, and SL ^ S ^ I (where S is sustainability, SL is social learning, and I is innovation) was used in this review (Fig. 2). Of the 63 studies selected, 40 addressed the intersection between sustainability and social learning, 18 handled the connection between innovation and social learning, and 5 were in the relationship between SL, S, and I. We chose these connections because the research approach addresses them, as shown in the theoretical framework. Hence, this systematic literature review adopts the same relations to support the objectives of this article.

There is a considerable number of studies on the intersection S ^ SL (n = 40), 18 papers on SL ^ I, and 5 articles on SL ^ S ^ I, highlighting how the literature needs to establish relationships between innovation, sustainability, and social learning. To investigate future research opportunities, we interpreted the contents of the 63 selected articles with the Atlas.ti software (Table 1).

**Table 1:** Article coding and distribution according to the frequency.

	Codes	Frequency
1	Collective action	613
2	Stakeholders	595
3	Beliefs and values	300
4	Changes	282
5	Context	254
6	Problem solving	222
7	Learning loops	196
8	Power	194
9	Behavior imitation	186
10	Cognition	135
11	Consensus	96
12	Participative processes	87
13	Communication	75
	**Total | accumulated**	**3235**

The frequency indicates the words or expressions that emerge from the selected papers through the software, making it possible to create codes, which can be marked at the exact point where they appear in the text under analysis. Repeating the codes, the software surveys the frequency in which they appear; then, we established a hermeneutic unit of analysis where the 63 articles appear continuously, allowing code markings in any of them. Therefore, we went back and forth in the articles, created codes between papers, and counted the code frequency uniformly within the hermeneutic unit. Creating and structuring the codes listed in Table 1 relates to the theoretical reading of the researcher, which allowed new codes to be discovered as they emerged during the analysis and organizing and grouping in light of their approximation.

We created the codes considering the context in which they were inserted because we then could classify them even if the literal word of the code did not appear in a certain passage. This was exemplified in the study of Seddon [35]: “It also comes across lived lives where social learning is reproduced through the reconstruction of social institutions, knowledge cultures, and governance practices,” which was marked with the code “stakeholders” for presenting a set of actors with interest in the subject addressed by the authors. Although the literal expression is absent, one can observe the context and meaning.

This coding form, which was carried out by an individual instead of automatic software, was the author's choice. Since this study is focused on social learning, sustainability, and innovation, automated surveys would certainly return the most frequently used words in spite of not mapping the meanings and contexts of the analyzed articles. Lastly, the following guiding questions (GQ) were established:

GQ 1 From what perspective is social learning approached?

GQ 2 How does the dialogue between social learning and innovation occur?

GQ 3 How does social learning appear in studies related to sustainability?

GQ 4 What future research proposals can be identified in terms of social learning, sustainability, and innovation?

## 3 Results and discussion

The results of this systematic literature review are presented below. To this end, the authors’ main conclusions were analyzed and distributed between social learning and innovation and social learning for sustainability; the analysis and discussion of the results are then presented.

### 3.1 Social learning and innovation

Authors who jointly addressed social learning and innovation in sustainability initiatives tend not to specify which innovation they refer to — whether social, technological, or sustainable innovation. In general, innovation seems to be treated as an umbrella concept characterized primarily by introducing a new or significantly improved good or service [36]. The authors and their findings are listed in Table 2, which focuses on the research conclusions and not the concept of innovation or social learning.

**Table 2:** Social learning and innovation for sustainability.

Source	Result
von Schönfeld et al. (2019) [37]	Innovation can appear through chaotic and non-inclusive means, in which social learning can be seen as a burden rather than an enabling process. There is a certain separation and independence between social learning and social innovation.
Hewlett (2021) [38]	i) Learning of innovations is horizontal rather than vertical, with individuals paying special attention to “successful” innovative models, usually adult men, preferably from outside the individual's social network; ii) teaching is an important process in transmitting innovations.
Beers et al. (2016) [39]	In innovation initiatives, the different types of interaction influence social learning. There are six types of interaction: antithetical interaction (debate of an idea that was accepted or rejected, where there are discussion and power struggles), synthetic interaction, information, power word, agenda wars, and conflict. Antithetical interaction tends to result in learning and impact as opposed to harmonious relationships, which gives a central role to conflict/dissent.
van Assche et al. (2013) [40]	The government plays a limited role in the social learning of innovations, as do officials or scientists. These actors had little influence on social learning results and emphasized product and service, institutional, and organizational innovations in rules and regulations.
Dessie et al. (2013) [41]	Social learning is a way to promote changes in rules and norms, creating opportunities for innovation. For example, changing views on the hierarchy between experts, farmers, and indigenous people and applying indigenous and scientific knowledge in the innovation process.
Sol et al. (2013) [42]	The presence of a facilitator is crucial in driving social learning processes; however, when the power to decide on resources and time is concentrated in this actor's hands, there can be a reduction in interaction and a sudden decline in mutual trust and commitment.
Boehmke and Witmer (2004) [43]	Innovation is diffused in two ways: social learning and economic competition (companies seeking to do better than competitors). Social learning influences adopting but not expanding, and economic competition should influence policy adopting and expanding.
Hinrichs et al. (2009) [44]	There is a contribution of social learning to marketing innovation in farmers' markets. Innovation is highly social and the ability of actors to learn within and between firms is key in the innovation process; this is because the focus is no longer on acquired knowledge but on the ability to learn since knowledge is changeable. Selling in the market more often increases the opportunity for social learning activities by giving more possibilities to interact with customers.
Aplin (2016) [45]	Social learning is used during sensitive periods of wild animal development, and social learning can provide behavioral responses to changing environments through the diffusion of innovations.
Miao (2017) [46]	Technological change and social learning contribute to disaster mitigation, particularly disasters that occur because of earthquakes. Countries with more disaster mitigation innovations and exposure to past earthquakes suffer fewer deaths.
Karubanga et al. (2017) [47]	The use of videos by farmers mediated by non-governmental organizations can trigger social learning by promoting farmers' access to information, innovations, and changes in agricultural practices. Videos trigger conversational exchanges among farmers, enabling collective reflection, evaluation, and validation of knowledge, motivating experimentation. Therefore, video documentation of farmers' innovation practices helps to broaden and deepen learning.
Garbach and Morgan (2017) [48]	Farmer networks are composed of contacts that can be pathways for social learning. Farmers' experiences related to management practices have positive and negative relationships with innovation adoption. Social learning was positively associated with support for the early stages of innovation adoption.
Barrantes and Yague (2015) [49]	Social learning processes can be the basis of an agricultural innovation method. New technologies in rural areas have failed due to a lack of interest from farmers, which led technicians to wonder if the method used to make technology transfer was not excluding the experience of farmers. There is a need for mutual respect for the knowledge of technicians and farmers. In this context, the “Working with People” approach, which consists of planning processes done by people and not for people, can help. Finally, culture and worldview matter, especially in innovation contexts.

The conclusions cannot be generalized in any of the studies in Table 2. However, research on the role of government and how learning influences innovation has shown that the authors’ conclusions in these specific studies are not unanimous, nor do they exclude any possibilities [37,40].

Various studies have demonstrated that social learning drives innovation [39,45,46,48,49]. Nonetheless, some researchers have reported that social learning can burden innovation processes [37], including conclusion is the exception among research on the topic. In general terms, the authors pointed to the driving role of social learning in innovation. These studies provide insights into what elements of social learning can drive innovation and why it places it in a prominent position in innovation projects. In other words, many of these studies seek to shed more light on how social learning can drive innovation.

The literature suggests that, in innovation contexts, if social learning occurs through the actions of individuals paying attention to innovative exogenous models in their network [38], considering antithetical interactions, such as debate of ideas as opposed to harmonious interaction, which is unfruitful for discussions [39], with the participation of public officials or scientists limited to the role of facilitator [40], and at the same time with an inclusive character in terms of participation of different actors [41], and through a balanced distribution of power in terms of decision about resources and time [44], the likelihood of innovation-oriented social learning is higher.

Social learning creates opportunities for innovation because it motivates change and questions rules and norms, the highly social quality of innovation, and the tendency to learn from actors — customers, suppliers, and employees, within and between firms [41,44]. Social learning contributes to teaching individuals how to learn continuously instead of accumulating information that is currently changeable and has the potential to provide behavioral responses to changing environments due to the ability to diffuse innovations [44].

Finally, social learning processes must happen by people and not by people [49]. Simultaneously to the contribution of social learning to innovation, many authors have pointed out that this theory plays an important role in the ideation, implementation, monitoring, and projection of sustainability. The following topic is an analysis of this research.

### 3.2 Social learning and sustainability

Studies on social learning for sustainability were the most representative. D'Angelo and collaborators [50] included ontological concerns that aim to analyze who individuals are becoming. According to the author, when analyzing how social learning for sustainability facilitates families from the base of the social pyramid (with no income) to change the condition of landless campers to family farmers with land moving up four levels in the social pyramid over a decade. From the farmers' narratives, it is possible to identify two social learning processes: ontological and epistemological [50]. In the first case, the authors analyzed learning dynamics under the questions of: Who learns? With whom do you learn? What do you learn? Where do you learn? When do you learn? Why do you learn? And how do you learn? In the second case, the understanding of whom the family farmers were becoming gained protagonism in the analysis. Table 3 shows the expressions that reflect the main ideas of the authors who have studied social learning for sustainability.

**Table 3:** Social learning for sustainability.

Source	Result
Fry and Thieme (2019) [51]	It is more helpful to share knowledge than to transfer information.
D'Angelo and Brunstein (2015) [52]	There is great relevance in learning together through experimentation.
Sol et al. (2013) [42]	The role of a facilitator is essential if they do not concentrate power on decision-making about resources and time, since it reduces interaction and trust.
Holden et al. (2014) [53]	When individuals are treated with empowerment, participation is higher.
Ofei-Manu and Shimano (2012) [54]	Schools play a leading role in promoting social learning to foster behavioral changes toward sustainability.
Johnson et al. (2012) [55]	Creating future scenarios through participatory workshops to stimulate social learning is a strategy that improves relationships by compressing the perspectives of others and creating foresight, as well as assisting in building new perspectives.
Garmendia and Stagl (2010) [56]	Workshops are a positive experience to increase comprehension of complex topics, such as environmental issues.
Wals and Rodela (2014) [57]	Sustainability is a continuous learning pursuit that may never be achieved, demanding a multidisciplinary debate.
Gottschick (2008) [58]	The interaction between scientists and politicians as a complex social system can be improved through social learning.
Luks and Siebenhuner (2007) [59]	Social learning for sustainability demands transdisciplinary knowledge, information from the social and natural sciences, and from common sense involving scientists and non-scientists.
Bouwen (2004) [60]	In water and soil management, the stakeholders should intervene in the planning and be responsible for the results in a conception of shared decision-making.
Siebenhüner (2004) [61]	Stakeholder participation is a way to integrate municipalities, interest groups, industry, and environmental groups to generate knowledge. The results of participatory procedures have greater legitimacy than those obtained under scientific knowledge produced within research institutions.
De Kraker (2021) [62]	The use of games favors the emergence of social learning on water management issues, at least in the cognitive dimension of learning (it concerns the perspectives of the problem, the solution, and the role of the self in the process). The games contributed to this shift from an individual to a group perspective.
Dlouhá et al. (2013) [63]	Social learning is a process that can be supported or driven by educational institutions, such as universities, and action-oriented.
Bolmsten and Kitada (2020) [64]	The social learning approach guides teaching methods for students to learn about the different spheres of sustainable development.
Sharma and Rani (2016) [65]	Social learning is used to inspire classroom teachers to use active and passive methodologies for teaching sustainability.
Seddon (2016) [35]	Social learning emerges from disruptive contexts and contributes to changing culture toward sustainable practices to resist changes in governance.
Hovardas (2021) [66]	Three tools can direct social learning to support the quest for social sustainability: the SWOT Matrix, the mixed motivation model (the search for convergence considering different interests, where stakeholders face tradeoffs but persist for the benefits of change/innovation), and tools involving participatory scenarios — these serve to guide stakeholder collaboration.
McGregor (2009) [67]	Social learning can reorient consumer education so that consumers change their behaviors to pursue sustainability.
Didham et al. (2017) [68]	Social learning facilitates collective engagement processes to achieve individual and collective learning for sustainable development. Cooperation and participation in decision-making solidify the conditions for a learning community.
Phuong et al. (2019) [69]	In terms of social learning for sustainability, three types of learning can be identified: instrumental (acquiring ecological, social, or economic knowledge, legal and administrative procedures), communicative (understanding what someone means, including oneself), and emancipatory (focuses on active dialogue to establish common goals and a joint action plan to make changes).
Kamaruddin and Pawson (2013) [70]	Looking locally can help young people learn socially, with the involvement of non-governmental organizations concerned with the fate of solid waste.
Pahl-Wostl (2002) [71]	1) Social learning is based on critical self-reflection, developing participatory processes, reflective capacities of individuals and societies, and the ability of social movements to shape political and economic conditions; 2) Social learning is seen as more important than decision-making based on factual knowledge; 3) The theoretical and conceptual basis of social learning and adaptive management is still weak and fragmented.
Lamboll et al. (2021) [72]	Multi-stakeholder social learning processes promote changes in key individuals' mindsets, norms, and values and offer an innovative approach to improving national-level policy and investment decision-making in sustainable agricultural systems.
D'Angelo et al. (2021) [50]	The existential ontological perspective can contribute to social learning theory for sustainability since much of the studies on social learning focus on the epistemological perspective.
Rekola and Riikka (2018) [73]	Social learning — based on social action, interactive reflection, communication, and negotiation, emerges as an alternative support method for transitioning to a more sustainable land use model. In the case under study, there were conflicts at the time of planning because those responsible rejected the opinions of other actors. The learning process was slow and did not turn into a substantial change in the interpretation of the planning process.
Axelsson et al. (2013) [74]	For social learning, a neutral facilitator is often needed to help the stakeholders; furthermore, there will be benefits if sectors of different levels and interests are included and have people with different backgrounds.
Médema et al. (2015) [75]	On multi-loop social learning in watershed management projects, the success of change is determined by the fit between four change factors that are interconnected, namely: 1) content factors: what is being changed or the type of change that is being implemented; 2) context factors: divided into external and internal factors of pre-existing forces and conditions that impact the effectiveness of the system; 3) process factors: how the change process is introduced and guided and how cooperation and decision-making are organized, with participation as a central factor; and 4) individual attributes: the idea that characteristics of those involved and those facilitating the change - also called change agents - impact reactions to change. Finally, it was noted that one challenge is to include much broader and more diverse groups of actors and stakeholders.
Hakansson et al. (2019) [76]	Social learning must change political hegemony to achieve social transformation rather than working towards behavior change (i.e., breaking reproductive patterns). There are four political aspects: political as inclusion and consensus, political as including cognitive and emotional elements, political as power relations, and political as part of decision-making processes.
Westoby and Lyons (2017) [77]	The key ingredient for successful transformative social learning in a politicized and dangerous context is building a social and relational network of people and organizations. Moreover, organizing communities together can give individuals the courage to fight against migration, one of the concerns highlighted in the Sustainable Development Goals. It is this network of organizations that creates the conditions for social learning. Without this network, people would be unable to think or imagine an alternative world and most certainly would not be able to organize social action. Social learning builds social movements.
Ison and Roeling (2007) [78]	Social learning involves a set of actions that encompass the collective but from a citizen's perspective. The concept still needs to be better understood and institutionalized — and it plays an important role in the training of a society.
Cincera et al. (2018) [79]	The results show that group diversity, participation, and contribution of other stakeholders, as well as social learning, can be understood as key characteristics of transdisciplinary knowledge alliances and play a crucial role in establishing the conditions for innovative and successful development of new curricula with a focus on sustainability, such as for sustainability-oriented entrepreneurship.
De Sousa (2021) [80]	Individuals participating in social learning processes use their different perspectives when reasoning and collaborating to solve environmental issues.
Rist et al. (2007) [81]	Sustainable development concepts must lose their normative character and acquire a set of ethical values that guide action resulting in changes in stakeholder interaction. Stakeholders need to be as diverse as possible to reflect reality. Social learning should be non-coercive, and its contents should be open to collective agreement, thus representing an “action-oriented philosophy” that is becoming increasingly prominent in European and developing countries. The focus of social learning is on the shift from multiple to collective cognition through the enlargement of spaces for social processes (the authors cite platforms or forums for deliberation, negotiation, and coordination of natural resource use as an example of this enlargement). Constructivism must rise above positivism. It demands spaces to transform strategic action into communicative action, which implies that actions are not coordinated based on a self-centered calculation of success (or strategic action), but through jointly defining situations relevant to action (or communicative action).
Schauppenlehner-Kloyber and Penker (2015) [82]	In projects where the social learning strategy was used, there was a deepening in the discussions, interactions, experiences, and cooperation process, and there was also an increase in trust between the government and population. Behavioral change results from social learning through group experiences and concerted action.
Rist et al. (2006) [83]	Social learning actors can be positively influenced by creating learning situations within social spaces involving different categories of actors within social processes. Social learning for sustainability should: build mutual trust, have less hierarchical communication patterns, and be grounded in the everyday life of local actors to change perceptions. After participating in workshops, participants may want to share the new information with their peers. Changes were noted in 1) interaction patterns, such as cooperation, changing attitudes, norms, and values 2) social competencies: social communication, sensitivity, conflict resolution 3) emotional competencies: empathy, expressiveness, intuition, and inspiration 4) cognitive competencies: increased awareness of the interrelationship of one's own and other participants' forms of knowledge and underlying ontologies and epistemologies were noted.

Table 3 allows us to map eight elements of social learning for sustainability.

1. *Stakeholder involvement.* According to social learning studies, the presence of stakeholders enables change if they are diverse and involved in the process [54]. The other steps are compromised without this involvement since these interactions will expose the experiences of those involved. Multi-stakeholder social learning processes promote changes in individuals' mindsets, norms, and values, involving actors despite the challenges [72,75]. Group diversity is critical in establishing the conditions for innovative development [79].
2. *Information sharing by multiple stakeholders.* Knowledge is built by multiple stakeholders rather than a process based on simply transferring [51]. The presence of different participants allows multidisciplinary debate, contributing to increasing trust and participation [57]. Another element that Kamaruddin et al. [70] highlight is focusing on the local by listening to multiple actors when aspects of the territory can be identified and reflect reality [81].
3. *Experimentation to learn together.* Practice is indispensable since the successes and mistakes of everyday life are responsible for changing standards, norms, and values.
4. *Acknowledge the valuing of all.* Discriminating and prioritizing one knowledge over another is a barrier to social learning [53]. The facilitator plays an important role by enabling shared decision-making and not concentrating power [42,60].
5. *Building common rules.* Stakeholders cooperate and shift toward sustainability when there is trust, rejecting the belief that there is no opportunity in relationships and that those involved seek the best for themselves. In this direction, their relationships can improve [55].
6. *Prospection of reality.* Research has acknowledged the relevance of collectively building hypothetical scenarios to visualize the consequences of present actions [55].
7. *Instrumentalizing social learning.* Many studies treat social learning instrumentally and list many tools that would lead to modifications towards a more sustainable world: universities and schools, workshops and meetings to discuss problems, games to reflect on environmental problems, teaching methods, and management tools [56,63,64,66]. However, there is still a lack of research exploring another dimension of learning, including how individuals have changed [50]. While some studies acknowledge that social learning promotes change, they do not indicate how it influences the actors involved [35].
8. *The emergence of conflicts.* If collective action of multiple stakeholders, social reflection, and cooperative participation are essential for social learning, these elements create conflicts that can undermine the process. Rekola and Riikka [73] reported evidence of this phenomenon and highlighted that conflicts in planning occurred due to the rejection of some opinions. Negotiation and communication can prevent conflicts from eroding behavior modification.

These elements are the basis for social learning to change political hegemony and achieve social transformation, pervading transitional behavior modification [76]. Emancipatory social learning enables one to build social movements by expanding spaces for social processes, from positivism to constructivism, which is strategic to communicate coordinated actions in an egocentric field for a cooperative definition [77,81].

Social learning for sustainability involves a set of attitudes, power dynamics, cooperation, and behavior change beyond the individual perspective [30,42,50,51,[53], [54], [55], [56],^58^,^84^,^85^]. The following section discusses the findings on social learning and innovation.

### 3.3 Analysis and discussion of the results

One of the focuses of this article is to investigate from which perspective social learning is envisioned (GQ 1: From which perspective is social learning approached?). Although the literature points to some connection of social learning with innovation in the field of sustainability studies, there is a rather peculiar perspective in these studies, corroborating the literature [50]: social learning is instrumentalized, with little concern for describing who individuals are becoming. As studies have progressed on the topic, research that focuses on what it is, how it occurs, why it occurs, etc. has multiplied, to the detriment of studies that show ontological aspects of learning [50].

Within this perspective is a dialogue between the concept of social learning and innovation (GQ 2: How does the dialogue between social learning and innovation occur?). This is mainly because both definitions seek changes in the generic and narrow sense, with researchers questioning whether social learning drives innovation or vice versa. One example is the study by von Schönfeld and Tan [37], who criticized the concept and accused it of hindering innovation precisely because of the methodical nature of social learning. Notably, it is unclear in the literature whether it is social learning that possesses this characteristic or whether it is research and actual case records that have assigned this epistemological connotation to the theory. Nevertheless, in summary, the dialogue between both concepts is that learning drives innovation [39,45,46,48,49].

Another relevant factor is that innovation can contribute to social learning, as if there were feedback processes. This was the conclusion of [47], in which the authors followed an innovation process by introducing videos teaching new management practices in a farming community. According to the authors, introducing this innovation broadened and deepened the learning of those involved partly because the videos were recorded and shared by the farmers themselves [47].

As in innovation research, social learning for sustainability is also treated from an instrumental perspective to achieve sustainability (GQ 3: How does social learning appear in studies related to sustainability?). This instrumental character is so evident that researchers have adopted the expression Social Learning for Sustainability, as if the theory was envisioned to serve sustainable issues. It is not that this cannot occur, although it is what is happening in the studies analyzed. In summary, we found little focus is given to ontological aspects to demonstrate who the parties involved have become after a given intervention using social learning.

**Fig. 3:**
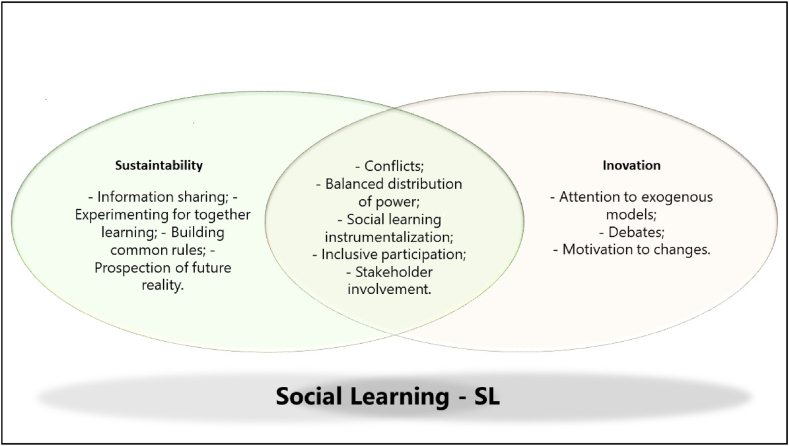
The intersection of Social Learning with Innovation and Sustainability.

The perspective attributed to social learning presents similarities of focus in studies on innovation and research focused on sustainability. The instrumental perspective that researchers have call the epistemological aspect predominates to the detriment of the ontological view [50]. The similarities contribute to the emergence of a research agenda addressing social learning, sustainability, and innovation (GQ 4: What future research proposals can be identified in terms of social learning, sustainability, and innovation?). There was an intersection in the approaches regarding social learning, which appeared in studies emphasizing innovation and sustainability (Fig. 3).

S**ource**: Survey data.

We found different terms in studies on social learning, such as those in Fig. 3, aligned with the theoretical concept [9,11,13]. Some of them appear in studies emphasizing innovation and when the focus is on sustainability (e.g., conflict existence, social learning instrumentalizing, and stakeholder involvement). Other terms are more frequent as they relate to sustainability-oriented studies, including information sharing and prospection of a future reality, or to innovation-oriented papers, including motivation to change, which is more common, although not prevalent, in innovation since the concept brings the idea of new practices [14,20].

Nevertheless, social learning is presented as a driver of both innovation and sustainability, and the innovation sought in the analyzed studies aims at implementing sustainability. For example, one study examined how adolescents in two hunter-gatherer communities in southwestern Ethiopia acquire and transmit knowledge related to environmental management [38]. In addition, even when social learning was instrumentally used to promote innovation, the purpose was to develop sustainability. Therefore, there is some interaction between social learning, innovation, and sustainability, which is an exciting avenue of research for future studies to confirm such existence and uncover how it occurs.

Another opportunity involves investigating who the actors participating in interventions instrumentalized by social learning are becoming, and this gap in the literature was also described in the literature [50]. Moreover, researchers can also investigate which elements of social learning are more explored when emphasizing innovation or sustainability. From the perspective of innovation, it is essential to demonstrate which one the studies refer to — whether social, technological, or sustainable. Finally, social learning for sustainability can occur through innovation, although it is unclear what within the concept of innovation facilitates social learning, which is seen as a behavioral change.

## 4 Conclusion

This study sought to identify future research directions on social learning, sustainability, and innovation from a systematic literature review. We observed some gaps around the possibility of studying social learning from an ontological perspective and for research demonstrating the intersections between social learning, innovation, and sustainability. Future studies could also indicate which elements of social learning are being explored (sustainability or innovation) and which features of social learning and innovation promote sustainability.

One of the strengths of this study is that the article uses a methodical strategy to perform a literature review by exploring several databases and establishing linkages between publications using hermeneutic units. The manuscript discusses a gap in the intersection of two theoretical strands—social learning and innovation — within the intricate sustainability framework. This fusion shed important light on how these ideas are related.

As a limitation, we highlight the concern with the innovation typology — whether social, technological, or sustainable innovation. Despite the terms used in the databases having filtered papers related to sustainability, the analysis could have focused, for instance, on articles that adopted a social perspective.

## Funding

This study did not receive any specific grant from funding agencies in the public, commercial, or not-for-profit sectors.

## Data availability statement

The data that support the findings of this study are available on request from the corresponding author.

## CRediT authorship contribution statement

**Jean Marcos da Silva:** Writing – review & editing, Writing – original draft, Visualization, Software, Project administration, Methodology, Investigation, Formal analysis, Conceptualization. **Jordana Marques Kneippb:** Writing – review & editing, Supervision, Software, Methodology, Conceptualization.

## Declaration of competing interest

The authors declare that they have no known competing financial interests or personal relationships that could have appeared to influence the work reported in this paper.
